# *Sloanea chocoana* and *S. pittieriana* (Elaeocarpaceae): Chemical and Biological Studies of Ethanolic Extracts and Skincare Properties

**DOI:** 10.3390/plants12233953

**Published:** 2023-11-24

**Authors:** Patricia Quintero-Rincón, Nayive Pino-Benítez, Elkin Galeano, Cris Rojo-Uribe, Ana C. Mesa-Arango, Oscar A. Flórez-Acosta

**Affiliations:** 1 Natural Products Group, Universidad Tecnológica del Chocó, Quibdo 270002, Colombia; patriciaquintero@gmail.com; 2 Research Group Design and Formulation of Medicines, Cosmetics, and Related, Faculty of Pharmaceutical and Food Sciences, Universidad de Antioquia, Medellin 050010, Colombia; oscar.florez@udea.edu.co; 3 Bioactive Substances Research Group, Faculty of Pharmaceutical and Food Sciences, Universidad de Antioquia, Medellin 050010, Colombia; elkin.galeano@udea.edu.co; 4 Dermatological Research Group, Faculty of Medicine, Universidad de Antioquia, Medellin 050010, Colombia; cris.rojo@udea.edu.co (C.R.-U.); ana.mesa@udea.edu.co (A.C.M.-A.)

**Keywords:** biodiversity and sustainability, bioprospecting, traditional medicine, genus *Sloanea*, skincare, human skin cells, antioxidant, UV protection, antifungal

## Abstract

The Colombian Chocó is known for its rich biodiversity and to harbor plant species that are under-explored, including the genus *Sloanea*. This study aimed to analyze the chemical composition of derivatized ethanolic extracts from *S. chocoana* and *S. pittieriana* using BSTFA and TMCS through GC–MS, and to assess cell viability of immortalized human non-tumorigenic keratinocytes (HaCaT) and periodontal ligament fibroblast cells using crude extracts through MTS assay. Antioxidant and photoprotective properties were determined using DPPH assay and spectrophotometry. Antifungal activity of extracts against *Candida* species was developed following the CLSI standard M27, 4th ed. The sun protective factor (SPF) and UVA/UVB ratio values were calculated using the Mansur equation and the Boots star rating system. The critical wavelength (λc) was determined by calculating the integrated optical density curve’s area. The transmission of erythema and pigmentation was calculated through equations that use constants to calculate the flux of erythema and pigmentation. The GC–MS analysis identified 37 compounds for *S. chocoana* and 38 for *S. pittieriana*, including alkaloids, triterpenoids, and polyphenolics, among others. Both extracts exhibited proliferative effects on periodontal ligament fibroblasts, did not affect the viability of HaCaT cells, and showed excellent antioxidant activities (46.1% and 43.7%). Relevant antifungal activity was observed with *S. pittieriana* extract against *Candida albicans* (GM–MIC: 4 µg/mL), followed by *C. auris* and *C. glabrata* (GM–MIC: 32 µg/mL), while *S. chocoana* extract was active against *C. albicans* and *C. glabrata* (GM–MIC: 16 and 32 µg/mL, respectively). High SPF values (31.0 and 30.0), λc (393.98 and 337.81 nm), UVA/UVB ratio (1.5 and 1.2), and low percentage of transmission of erythema and pigmentation were determined for *S. chocoana* and *S. pittieriana*, respectively. Results showed that species of *Sloanea* constitute a promising alternative as ingredients for developing skincare products, and exhaustive studies are required for their sustainable uses.

## 1. Introduction

The genus *Sloanea* L. (Elaeocarpaceae) consists of approximately 185–200 species globally, with around 130 species found in the Neotropics [1,2]. This genus is characterized by trees with remarkable tabular roots, generally apetalous flowers, and fruits with rigid or flexible spines, which can be irritating. Neotropical *Sloanea* species are distributed from Mexico to southern Brazil and certain Caribbean islands [3]. Among the 38 species in the Neotropical section Brevispicae, 12 can be found in the Colombian Chocó [4], including *S. garcia-cossioi* [5], *S. calva* [6], *S. pacuritana* [7], *S. esmeraldana* [8], *S. chocoana* [9], *S. durissima*, *S. guianensis*, *S. laevigata*, *S. laxiflora*, *S. macrophylla* [10], *S. helianthoides*, and *S. huilaeana* [4]. These species are generally called “achiotillo” by local communities of Chocó and are used traditionally to treat malaria, fever, and inflammation [11].

Although ethnobotanical studies have provided significant information on the taxonomy and distribution of *Sloanea* species, knowledge about the chemical composition and biological effects of this genus is under-explored [4,6,8,12]. However, a recent study highlighted the biological activity of two extracts obtained from wild species of *Sloanea*, emphasizing their skincare properties. The study assessed the use of ethanolic extracts of *S. medusula* and *S. calva* in the development of extract-based gels, aiming to provide an alternative treatment for cutaneous candidiasis with antioxidant and UV-protective effects [13]. 

In this research, the chemical and biological characteristics of two species, including an autochthonal species from the Colombian Pacific region are analyzed. *Sloanea chocoana* Pal.–Duque is a 10 m tall tree whose specific epithet refers to Chocó, the Colombian department where the species was first identified [9,12]. *Sloanea pittieriana* Steyerm. is a tree named after the botanist Henry Pittier, growing up to 30 m high and thriving at very low altitudes, between 50 and 100 m.a.s.l., on alluvial soils [14]. 

The study of plants, extracts, essential oils, and derivatives for medicinal purposes is an important topic for the global governments [15,16]. In Latin-American countries such as Colombia, some strategies have been established to ensure the sustainable use of these natural sources [17,18], mainly because this knowledge can help achieve the United Nations Sustainable Development Goals, particularly Goals 1 (No Poverty), 3 (Good Health and Well-Being), and 12 (Responsible Consumption and Production) (https://www.un.org/sustainabledevelopment/sustainable-development-goals/ accessed on 25 January 2023).

Extracts and essential oils obtained from plants are valuable sources of compounds with antioxidant properties, including alkaloids, flavonoids, phenols, stilbenes, triterpenes, tannins, and carotenoids, which act by inhibiting damage caused by reactive oxygen species (ROS)-induced oxidative stress; among them are the hydroxyl (HO^•^), super-oxide (O_2_^•·−^), peroxyl (RO_2_^•^), alkoxyl (RO^•^), and singlet oxygen (^1^O_2_) radicals, as well as hydrogen peroxide (H_2_O_2_) and organic peroxides (ROOH) [19,20].

The skin, as an external organ, can eventually be damaged by diverse environmental and biological factors, including the aging process, cumulative exposure to UV radiation [21,22], and alterations of its normal flora [23]. In this sense, natural antioxidants obtained from plants provide benefits, especially for skincare [24]. Diverse species have been demonstrated to exhibit photoprotective [13,25,26], antibacterial [27,28,29], antifungal [13,30], antiviral [31], and anticancer properties [32,33]; therefore, it is crucial to deepen the knowledge of chemical and biological aspects of plant diversity.

Responding to the demand for new knowledge and valuation of the biodiversity of the Colombian Pacific flora, this study aimed to identify phytochemicals present in the ethanolic extracts of leaves from *S. chocoana* and *S. pittieriana* collected in Chocó, Colombia, and to evaluate their biological properties to determine their potential as wound healing, antioxidant, photoprotective, and antifungal agents.

## 2. Results

### 2.1. Chemical Studies 

#### Gas Chromatography–Mass Spectrometry (GC–MS) Analysis

The GC–MS analysis identified a total of 37 compounds for *S. chocoana* (Table 1) and 38 compounds for *S. pittieriana* (Table 2). These compounds accounted for more than 50% and 60% of the total content of each derivatized ethanolic extract, respectively.

### 2.2. Biological Studies

#### 2.2.1. Evaluation of the Viability of Immortalized Human Non-Tumorigenic Keratinocytes (HaCaT) and Periodontal Ligament Fibroblast Cells

**Figure 1:**
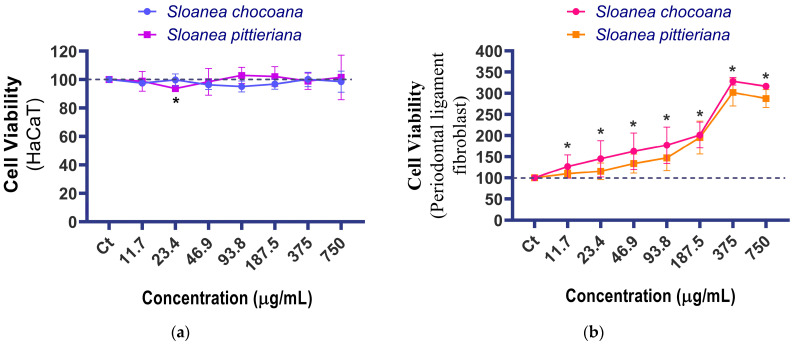
Viability assay of cells exposed to ethanolic extracts from *S. chocoana* and *S. pittieriana:* (**a**) on an immortalized human non-tumorigenic keratinocyte (HaCaT) cell line, (**b**) on a periodontal ligament fibroblast cell line. * Significant difference (*p* < 0.05).

The influence of *S. chocoana* and *S. pittieriana* extracts on cell viability of HaCaT and periodontal ligament fibroblast cells was quantified using the MTS assay after 24 h of exposition. Both extracts exerted similar effects across the concentration range evaluated (11.7 to 750 μg/mL) (Figure 1a,b). Notably, neither cell type experienced any cytotoxic effects, but there was a significant and noteworthy increase in periodontal ligament fibroblast cell proliferation, starting at a concentration of 11.7 μg/mL (Figure 1b). The maximum cell proliferation was observed at 375 μg/mL and remained consistently elevated until the highest concentration (750 μg/mL).

#### 2.2.2. Evaluation of the Antifungal Activity

To determine the minimum inhibitory concentration (MIC), the extracts were evaluated at ten concentrations ranging from 0.5 to 256 μg/mL against *C. albicans* ATTC 10231, *C. auris* Ca17 (L25), *C. glabrata* LMDM 34 (L7), *C. krusei* ATCC 6258 (L6), and *C. tropicalis* ATCC 200956. The values expressed as the geometric mean (GM)-MIC are shown in Table 3. Laboratory reference *Candida* species and clinical isolates evaluated showed different antifungal susceptibility profiles. The antifungal activity of *S. chocoana* extract was most remarkable against *C. albicans* (GM–MIC of 16 μg/mL). In contrast, *S. pittieriana* extract was most active against *C. albicans* (GM–MIC of 4 μg/mL) and *C. auris* (GM–MIC of 32 μg/mL). Additionally, *S. pittieriana* extract showed an antifungal potential similar to *S. chocoana* extract against *C. glabrata* (GM–MIC of 32 μg/mL).

#### 2.2.3. Evaluation of the Antioxidant Activity

The ethanolic extracts from *S. chocoana* and *S. pittieriana* were evaluated to determine their antioxidant properties based on their ability to eliminate free radicals from DPPH and their antiradical efficacy. Table 4 shows the antioxidant potential of both extracts in terms of DPPH radical scavenging activity expressed in percentage and Trolox equivalent antioxidant capacity (TEAC). It is observed that ethanolic extracts from *S. chocoana* and *S. pittieriana* exhibit excellent antioxidant capacity. *Sloanea chocoana* is remarkable due to its DPPH radical scavenging activity (46.1%) and TEAC (3.7 mM Eq. Trolox/100 g sample weight) in comparison with *S. pittieriana*, whose values are 43.7% and 2.0 mM Eq. Trolox/100 g sample weight, respectively.

#### 2.2.4. Determination of Photoprotective Properties

The in vitro photoprotection indices were estimated using spectrophotometric analysis. A concentration of 0.75 mg/mL of the extract was employed in the data acquisition process. Table 5 shows the potential of extracts from *S. chocoana* and *S. pittieriana* as sunscreens. In this sense, both extracts provide high protection against ultraviolet (UV) radiation: *S. chocoana* (SPF 31) and *S. pittieriana* (SPF 30). A critical wavelength of 393.98 nm was determined for *S. chocoana* extract, indicating that this extract could act as UVA long-wavelength sunscreen similar to commercial sunscreen, while *S. pittieriana* offers UVB protection. UVA/UVB ratio (1.5 and 1.2), erythema transmission (0.0 and 0.0%), and pigmentation transmission (0.0 and 2.8%) indicate that both extracts offer broad protection against UV rays, are effective in preventing the appearance of redness (erythema), and have the ability to prevent the appearance of dark spots (pigmentation) on the skin after exposure to UV radiation, respectively.

## 3. Discussion

The Colombian flora has enormous potential as a source of global resources, due to its advantageous geographical position and water availability [34]. The diverse ecosystems found in this country, including tropical rainforests, play a vital role in the preservation of biodiversity and the advancement of sustainable development [35,36].

The Colombian Chocó is one of the wettest places on earth [37] and is remarkable for hosting a rich variety of plant species with the potential for implementing a bioeconomic model [38]. Despite its relevance, the flora of Chocó has been under-explored, and studies of prospecting for different plant genera, including *Sloanea,* are limited.

Currently, the chemical characterization and biological properties of four species of the genus *Sloanea* have been reported. In Colombia, leaf extracts of *S. medusula* and *S. calva* were studied as antioxidant, photoprotective, and anti-*Candida* agents. These bioactivities were attributed to hydrolyzed tannins and pentacyclic triterpenes identified in the extracts through HPLC–MS/MS [13]. In Panama, cytotoxic properties on human cancer cell lines of the breast, lung, and central nervous system were identified employing triterpenoids isolated from *S. zuliaensis* [39], and in Madagascar, antiplasmodial properties have been identified using polyphenols obtained from *S. rhodantha* [40].

In this study, the GC–MS analysis led to the identification of silylated sugars, fatty acids, and biosynthetic derivatives (Table 1 and Table 2). Common silylated compounds identified in *S. chocoana* and *S. pittieriana* extracts were derived phenolic (gallic acid), polyol (myo-inositol), cyclic polyol (quinic acid), acyclic diterpene alcohol (phytol), flavan-3-ol (catechin), tetracyclic triterpenoids (stigmasterol and (3b,24S)-stigmast-5-en-3-ol), pentacyclic triterpenoid (lupeol), and glycerol and its glycoside derivative (glyceryl glycoside). In addition, in the *S. chocoana* extract, we identified indole alkaloid ((3E)-5-ethyl-1H-indole-2,3-dione 3-oxime), phenolic (chlorogenic acid), quinic derivative (5-O-coumaroyl-D-quinic acid), the alpha-galactoside of inositol (galactinol), flavan-3-ol (epicatechin and epigallocatechin), and terpene phenol (tetrahydrocannabinolic acid) (Table 1). While indole alkaloid (cholest-2-eno[2,3-b]indole, 1′-methyl-5′-methoxy-), ketotriose (dihydroxyacetone), amine (2-amino-1-phenylethanol), tetracyclic triterpenes (campesterol and gamma-sitosterol), pentacyclic triterpenes (lup-20(29)-en-28-oic acid, alpha-amyrin, and oleanolic acid), and benzoic acid were identified in *S. pittieriana* extract (Table 2).

**Figure 2:**
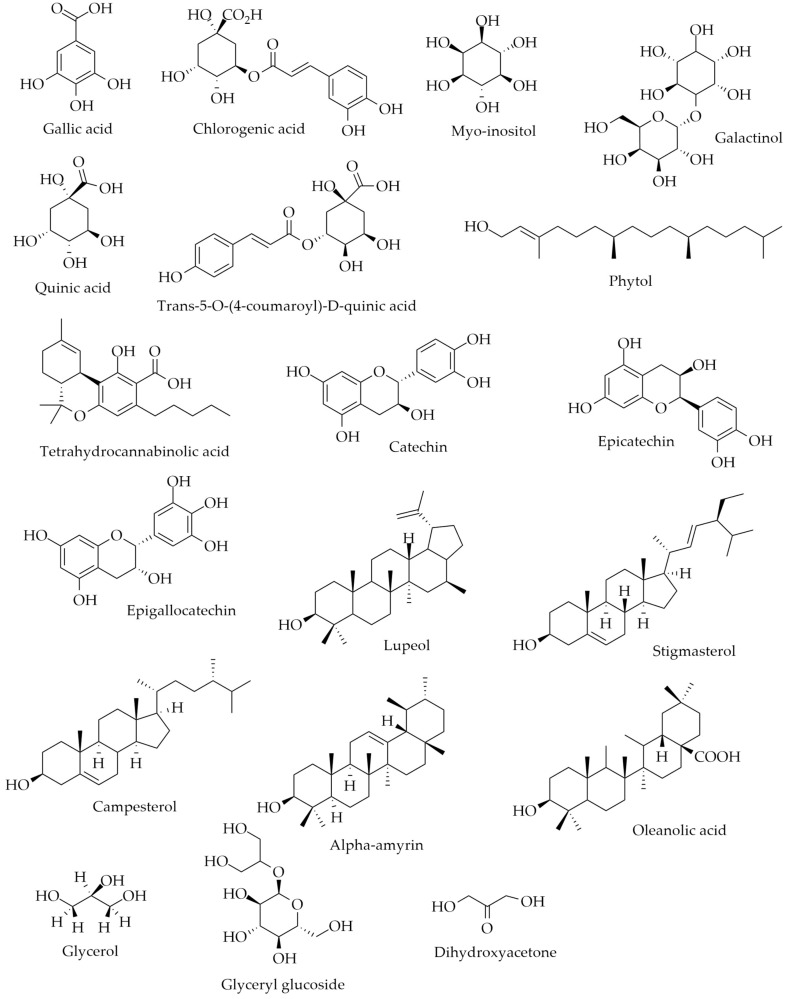
Chemical structures of potential molecules for the development of skincare products identified in *Sloanea chocoana* and *S. pittieriana* extracts.

A literature review allowed us to correlate the compounds identified in the extracts with their biological activities. In this sense, gallic acid (a polyhydroxyphenol compound) and the esters of caffeic acid and quinic acid (chlorogenic acid) have been identified as antioxidants and anti-inflammatory agents [41,42]. Gallic acid is currently studied due to its anticancer properties [41], while chlorogenic acid has remarkable metabolic regulatory features [42] and is a potential botanical insecticide [43]. Myo-inositol (a cyclic polyalcohol) present in plant species, e.g., the Cactaceae family, contributes to overcoming the intracellular deficiencies of inositol and ameliorating the adverse effects caused by its abnormal metabolism, notable in patients at risk of diabetes or insulin resistance and cancer patients [44]. Galactinol or 1-O-alpha-D-galactopyranosyl-L-myo-inositol (plant defense signal), plays a role in the biosynthesis of the raffinose family of oligosaccharides, which are found in plant species and serve as desiccation protectants in seeds and transport sugars [45]. Quinic acid, a cyclohexanecarboxylic acid present in plant species from the Asteraceae, Hypericaceae, Menispermaceae, Polygonaceae, and Rubiaceae families, has been reported for its antioxidant, protective, antidiabetic, antimicrobial, anticancer, antinociceptive, analgesic [46], and antiaging effects [47]. In silico analysis employing the natural product trans-5-O-(4-coumaroyl)-D-quinic acid showed a binding affinity with SARS-CoV-2 main protease (MPro; PDB ID: 6LU7) and mediates viral entry in the host suggesting its antiviral activity [48]. Phytol is an acyclic alcohol diterpenic with antioxidant, anti-inflammatory, antimicrobial, anxiolytic, metabolism-modulating, immune-modulating, anticancer, and antinociceptive properties [49]. Tetrahydrocannabinolic acid is a phytocannabinoid with neuroprotective and neuroinflammatory effects [50]. Flavanols, including catechin, epicatechin, and epigallocatechin are polyphenols recognized for exerting antioxidant effects [51]. These phytocompounds have been used by the cosmeceutical industry in extract-based nanoformulations due to their properties as sunscreen, and moisturizing and antiaging agents [52]. Tetracyclic and pentacyclic triterpenoids are phytocompounds extensively studied as wound healing agents [53]. The antioxidant, anti-inflammatory, antidiabetic, anticancer, and hepatoprotective effects of lupeol are amply studied [54]. Phytosterols such as stigmasterol and campesterol exert metabolic effects [55], and possess antioxidant, antimicrobial, and photoprotective properties [56]. Gamma-sitosterol is a natural product with antioxidant, antimicrobial, antidiabetic, and photoprotective properties [56,57]. Alpha-amyrin is a natural product derived from ursane with antibiofilm properties against methicillin-resistant and vancomycin intermediate-resistant *Staphylococcus aureus* [58]. Oleanolic acid exerts anti-inflammatory, anticarcinogenic, antimicrobial, antiatherosclerotic, hypolipidemic, hepatoprotective, and gastroprotective properties [59]. Glycerol is used in skin care products because acts as an emollient and prevents skin lesions [60], while glyceryl glycoside is used as an emollient in formulations for the treatment of psoriasis [61]. Dihydroxyacetone is used as an additive in bronzers and spray tan booths [62], and benzoic acid is used as a preservative in the cosmetic and food industries [63,64]. The chemical structures of some of these potential molecules are shown in Figure 2.

This study evaluated the biological potential of ethanolic extracts from *S. chocoana* and *S. pittieriana* on HaCaT cells and periodontal ligament fibroblasts to demonstrate their possible applications in wound healing. An antiproliferative effect on HaCaT cells was not observed, which could support the use of these extracts in topical formulations [65]. On the other hand, the proliferative effect of both extracts observed in all tested concentrations on periodontal ligament fibroblasts suggests a potential application for the regeneration of periodontal tissues [66]. A study about the modulatory effects of extracts of *S. chocoana* and *S. pittieriana* on periodontal stress using these fibroblasts could lead to a broader understanding of periodontal health and contribute to the development of novel treatments for oral diseases [67,68].

The anti-*Candida* activity observed in the evaluated extracts suggests a potential to develop antifungal agents targeting various *Candida* strains, among them *C. albicans*, *C. auris* (considered critical fungi according to the WHO), and *C. glabrata* (high priority group) [69]. These extracts exhibit similar MIC values to other plant extracts recognized for their antimicrobial properties [70].

Although candidiasis of the skin is generally not life-threatening, it can cause significant skin alterations when the yeasts proliferate excessively [71]. Sometimes, these superficial infections can cause severe infections, persistent, and resistant to treatment, inducing a condition known as mucocutaneous candidiasis [72]. Therefore, strategies that lead to the development of new treatment alternatives for candidiasis are often a priority in research [13].

Regarding antioxidant and photoprotective activity, both ethanolic extracts exhibit good antioxidant activity, which could influence their ability to capture UV radiation and induce photoprotective properties [73]. Due to these benefits, *S. chocoana* and *S. pittieriana* extracts could be used as natural ingredients in formulations for cosmetic applications. Antioxidant properties of the phytocompounds are valuable in topical formulations because natural ingredients can help neutralize free radicals responsible for oxidative stress and skin damage [74]. Moreover, the photoprotective properties exhibited by *S. chocoana* extract indicate that it can protect the skin from damage caused by long-wavelength UVA radiation, which is relevant for formulating sustainable sunscreen for cosmetics [75].

Our results constitute the first scientific evidence that supports the use of *S. chocoana* and *S. pittieriana* in the practices of traditional medicine. Therefore, exhaustive prospecting studies of these *Sloanea* species are necessary to obtain a broader understanding of their biological potential.

## 4. Materials and Methods

### 4.1. Materials

All reagents and solvents were used without further purification. The commercial suppliers were: Sigma-Aldrich Co., St. Louise, MO, USA (amphotericin B, itraconazole, Trolox, Sodium acetate, Sabouraud dextrose agar, and RPMI 1640–MOPS). Sigma-Aldrich, Darmstadt, Germany (chlorotrimethylsilane, TMCS; pyridine anhydrous 99.8%). Sigma Chemical Co., St. Louise, MO, USA (DPPH reagent (1,1-diphenyl-2-picrylhydrazyl). Merck, Rahway, NJ, USA (methanol). Merck KGaA, Darmstadt, Germany (N-O-bis-trimethylsilyl-trifluoroacetamide, BSTFA). GIBCO^TM^, Paisley, UK (Dulbecco’s modified Eagle’s medium, DMEM; fetal bovine serum, FBS; penicillin 100 U/mL–streptomycin 100 µg/mL, P/S). GENFAR^®^, Bogotá, Colombia (gentamicin 100 U/mL). Promega Corporation, Madison, WI, USA (anionic tetrazolium salt known as 3-(4,5-dimethylthiazol-2-yl)-5-(3-carboxymethoxyphenyl)-2-(4-sulfophenyl)-2H-tetrazolium, MTS, Cell Titer 96^®^ AQ_ueous_ Non-Radioactive Cell Proliferation Assay). Corning^®^, Sommerville, MA, USA, and Costar^®^, Washington, DC, USA (96-well microdilution plates).

The HaCaT cells were provided by Prof. Ph.D. Belfran Carvonel from the Genetic, Regeneration, and Cancer Group (University of Antioquia). The periodontal ligament fibroblasts were derived from primary cultures of fibroblasts from a patient’s periodontal ligament (the study was approved by the research and ethics committee of the Faculty of Dentistry at Pontificia Universidad Javeriana through Act #005 of 12 February 2021).

### 4.2. Plant Material Collection and Extraction

**Figure 3:**
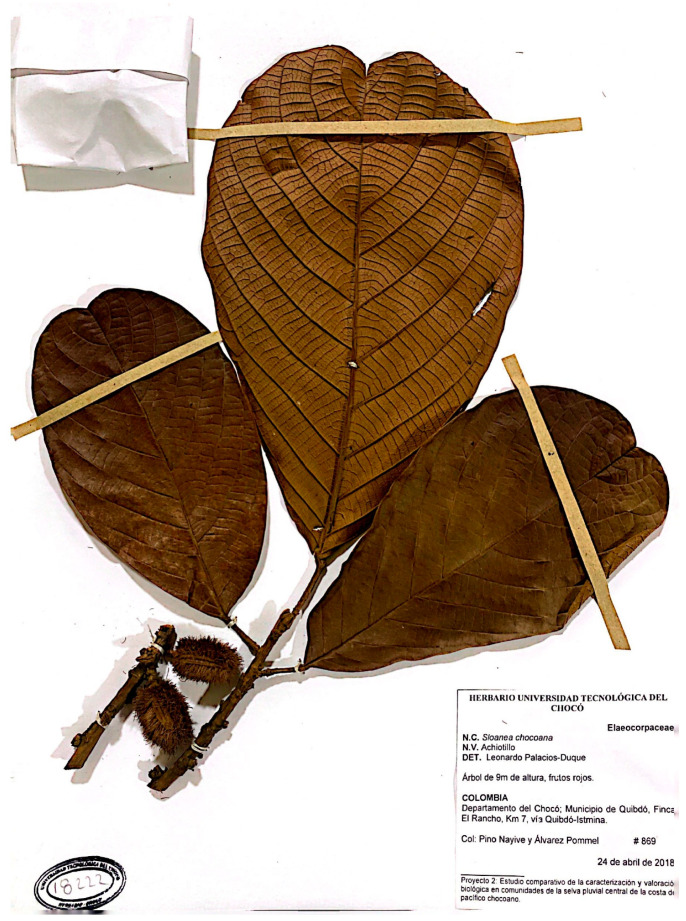
Botanical specimen of *Sloanea chocoana* Pal.-Duque.

**Figure 4:**
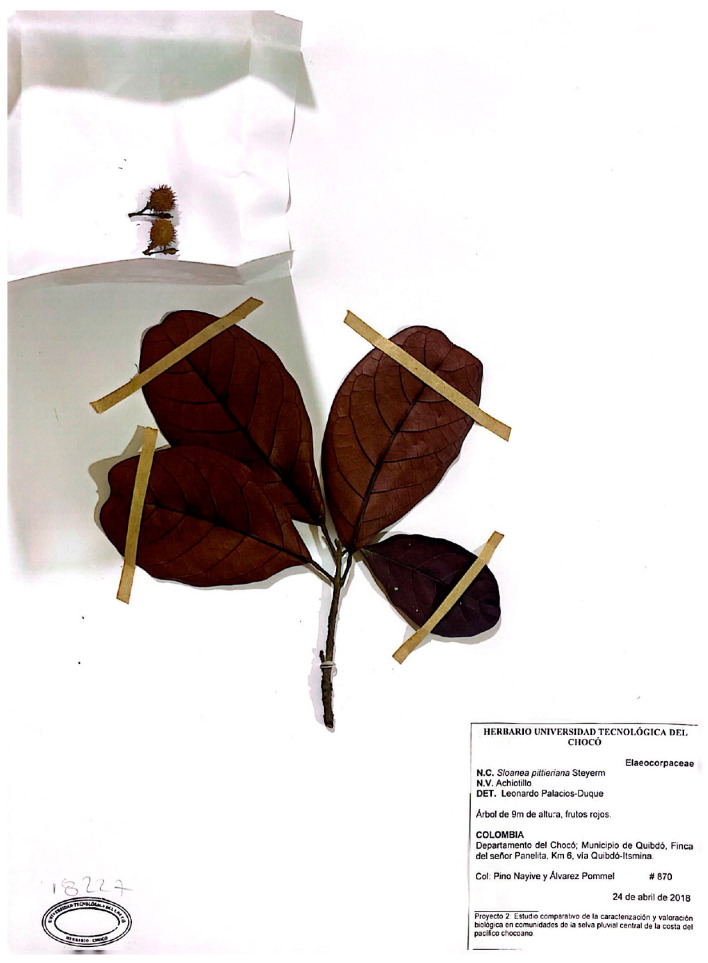
Botanical specimen of *Sloanea pittieriana* Steyermark.

Plant materials were collected in Quibdó, Chocó Department, Colombia: Farm El Rancho, Km 7, via Quibdó-Istmina (*Sloanea chocoana* Pal.-Duque), and Farm of Mr. Panelita, Km 6, via Quibdó-Istmina (*Sloanea pittieriana* Steyermark). The specimens were deposited in the Herbarium of the Technological University of Chocó (Quibdó), along with their corresponding vouchers: *S. chocoana* (869 Herbario CHOCÓ) (Figure 3), and *S. pittieriana* (870 Herbario CHOCÓ) (Figure 4). Leonardo Palacios-Duque, a specialist in *Sloanea* species at the Technological University of Chocó, identified the evaluated species.

Plant species were collected in accordance with resolution 1601 of 26 November 2015, which grants permission to collect specimens of wild species, issued by the relevant environmental authority CODECHOCÓ.

The extracts were prepared by subjecting the leaves to air drying, followed by a grinding procedure with a ball mill. Then, plant material was macerated in 96% ethanol for three days (without stirring). Subsequently, the ethanol was filtered, and the residue was extracted two-fold. The crude extract was obtained using rotary evaporation. The yield obtained relative to the fresh plants was 1.4% for *S. chocoana* and 1.3% for *S. pittieriana*. Finally, both extracts were appropriately labeled and kept at a temperature of 4 °C until they were ready to be used.

Ethanol is frequently used in traditional medicinal practices of Chocoan communities and was chosen and employed for extraction processes. Ethanol is known to effectively extract phytocompounds with desirable therapeutic properties, including polyphenolics and triterpenoids, among others [76].

### 4.3. Gas Chromatography–Mass Spectrometry Analysis

#### 4.3.1. Derivatization of Extracts

Initially, both extracts underwent analysis using GC–MS to characterize their volatile fraction. In this analysis, the identification of relevant compounds was not achieved, suggesting the presence of phytochemicals with high molecular weight and non-volatile nature, such as flavonoids, glycosides, phenolics, fatty acids, and steroids, among others. As a result, the crude extracts could not be analyzed using GC–MS. For this reason, the extracts were derivatized employing 0.05 mL of BSTFA + 1% TMCS and 0.05 mL of pyridine, with the purpose of changing the non-volatile nature of the compounds to volatile compounds, making them analyzable using GC–MS. The reaction was incubated for 2 h at 70 °C. The derivatized products were analyzed using GC–MS.

#### 4.3.2. Data Acquisition

The analysis using GC–MS was conducted employing an Agilent 7890 GC apparatus (Wilmington, DE, USA) equipped with a 5975C mass selective detector and a CTC Analytics PAL3 autosampler with a capillary column: HP–5MS (30 m, 0.25 mm i.d., film thickness 0.25 μm) (Agilent J&W). The oven temperature was programmed to start at 120 °C (5 min), increase to 180 °C at 7 °C/min (5 min), then increase by 10 °C/min to 200 °C for 5 min; after, the increase was 10 °C/min to 300 °C for 5 min, and finally to 310 °C at 10 °C/min (5 min). The injector temperature was set at 290 °C in splitless mode, and 1 μL of sample was injected. The mass selective detector temperatures of the ionization chamber and MS Quad were set at 230 and 150 °C, respectively. All mass spectra and total ion current (TIC) chromatograms were obtained using automatic scanning at 4.51 scan^−1^. The electron impact ionization energy was 70 eV, and the mass range was *m*/*z* 45–850.

#### 4.3.3. Identification of the Compounds

Mass spectra of the compounds found in the extracts were matched with data in the Mass Spectral Library of the National Institute of Standards and Technology (NIST) 17 [77].

### 4.4. Cell Viability Assay

HaCaT and periodontal ligament fibroblast cells were cultivated in DMEM supplemented with 10% SBF, 1% penicillin (100 U/mL)–streptomycin (100 µg/mL), and gentamicin (100 U/mL) at 37 °C (5% CO_2_). The assessment of cell viability was performed using MTS tetrazolium salt following a protocol described by Loaiza-Oliva et al. [78] with minor modifications. This salt requires an electron-coupling reagent to penetrate live cells. When active dehydrogenase enzymes are present, the colorimetric salt permeates the cytosol of metabolically active cells, undergoes reduction, and forms an intracellular purple precipitate called formazan. Formazan quantification can be achieved by measuring absorbance using a spectrophotometer or a plate reader [79].

For viability evaluation, a total of 1.5 × 10^4^ cells per well were seeded in 96-well plates and were incubated at 37 °C (5% CO_2_) until 80% confluence. The medium was removed and serial 1:2 dilutions of the extracts were added, resulting in seven concentrations ranging from 11.7 to 750 μg/mL. Then, the cells were incubated for 24 h at 37 °C (5% CO_2_). The serial dilutions were removed, and each well was treated with 100 µL of DMEM and 20 µL of MTS. After incubation (3 h), the absorbance was measured at 493 nm using a Multimode Microplate Reader (Thermo Fisher Scientific, Inc., Waltham, MA, USA). The CC_50_ values were determined through linear regression analysis using the GraphPad Prism 8.0.1 (244) program (San Diego, CA, USA) based on two independent assays conducted in triplicate. Significance was set at a *p*-value less than 0.05.

### 4.5. Antifungal Activity Assay

For the antifungal activity assay, we used three laboratory reference strains (*C. krusei* ATCC 6258, *C. albicans* ATCC 10231, and *C. tropicalis* ATCC 200956) and two clinical isolates (*C. auris* Ca17 (L25) and *C. glabrata* LMDM 34 (L7)). All yeasts were cultured on Sabouraud dextrose agar for 24 h (at 35 °C). The antifungal susceptibility testing was performed according to the Clinical and Laboratory Standards Institute (CLSI) standard M27, 4th edition [80]. The evaluation of itraconazole and amphotericin B activity against *C. krusei* ATCC 6258 [81] was used as technical control where the MIC values needed to remain within the accepted range: itraconazole (0.125 μg/mL) and amphotericin B (0.630 μg/mL). To evaluate the anti-*Candida* activity, the following steps were performed: (1) Stock solutions of extracts at the concentration of 512 µg/mL and an inoculum of 2.5 × 10^3^ CFU/mL in RPMI 1640–MOPS of each yeast were prepared separately. (2) The stock solution of each extract (100 µL) was added to 96-well microdilution plates, followed by yeast inoculum (100 µL). Under these conditions, 10 two-fold dilutions of each extract ranging from 0.5 to 256 µg/mL and a concentration of the inoculum at 1.25 × 10^3^ CFU/mL were evaluated. (3) The plates were incubated at 35 °C (24 h). MIC values were visually determined using a manual mirror viewer, and were considered as the lowest concentrations that produced visual inhibition compared to the growth control. The assays were performed on different days, at least three times in duplicate. Results are expressed as the GM–MIC.

### 4.6. DPPH Assay

This study aimed to assess the hydrogen atom-donating ability of extracts obtained from *S. chocoana* and *S. pittieriana* by measuring the decolorization of a solution of DPPH. The methanolic solution of DPPH changes from violet/purple to shades of yellow in the presence of compound antioxidants [82]. The DPPH radical scavenging assay was conducted following a methodology previously reported [13]. Results are expressed as the mean ± standard error from three independent assays.

### 4.7. Evaluation of Photoprotective Activity

Absorbance measurements at different wavelengths, ranging between 290 and 400 nm, were taken to determine the SPF, λc, UVA/UVB ratio, and percentage of transmission of erythema and pigmentation, respectively, following methodologies employed in previous studies [13,25]. Criteria for consideration of in vitro photoprotective potential are shown in Supplementary Table S1.

## 5. Conclusions

In conclusion, the GC–MS analysis allowed a first approach to understanding the chemical nature of the extracts evaluated and their justification in traditional medicine. However, since this is the first report on the chemical characterization of *S. chocoana* and *S. pittieriana*, it is suggested to perform exhaustive investigations to get to know the nature of the phytocompounds present in both extracts that can be used for developing drug or skincare products.

Cell viability results indicate that *S. chocoana* and *S. pittieriana* extracts did not exhibit toxicity against HaCaT cells and could be used in topical formulations. Furthermore, these extracts showed proliferative effects on periodontal ligament fibroblasts, indicating their potential for promoting cell growth in periodontal tissues and suggesting further investigation to understand the underlying mechanisms.

Extracts exhibited notable antioxidant and in vitro photoprotective activity, especially *S. chocoana*, which means they could be used as sunscreens. The antifungal activity observed in *S. pittieriana* against *C. albicans*, *C. auris*, and *C. glabrata*, indicates a potential to protect the skin from fungal infections.

These findings highlight the importance of studying under-explored natural sources to discover and offer sustainable and effective skincare ingredients.

## Figures and Tables

**Table 1:** Peak identification for the chromatogram of silylated *S. chocoana* extract, which is shown in Supplementary Figure S1.

No.	Silylated Derivative	Retention Time (RT, min)	Formula (DB)	Area	Relative Concentration (%)
1	Glycerol, 3TMS	7.33	C_12_H_32_O_3_Si_3_	287346350	1.8
2	meso-Erythritol, 4TMS	12.27	C_16_H_42_O_4_Si_4_	348625307	2.2
3	1H-Indole-2,3-dione, 5-ethyl-1-(trimethylsilyl)-, 3-[O-(trimethylsilyl)oxime]	13.49	C_16_H_26_N_2_O_2_Si_2_	43561683	0.3
4	beta-Lyxopyranose, 4TMS	14.87	C_17_H_42_O_5_Si_4_	26506888	0.2
5	L-(−)-Arabitol, 5TMS	16.87	C_20_H_52_O_5_Si_5_	110844089	0.7
6	1,3-Dihydroxyacetone dimer, 4TMS	17.45	C_18_H_44_O_6_Si_4_	177106325	1.1
7	L-(−)-Sorbofuranose, 5TMS	18.51	C_21_H_52_O_6_Si_5_	33148204	0.2
8	Quinic acid, 5TMS	20.86	C_22_H_52_O_6_Si_5_	722822995	4.7
9	D-Fructose, 5TMS	21.41	C_21_H_52_O_6_Si_5_	152605632	1.0
10	D-(+)-Galactopyranose, 5TMS (isomer 2)	21.79	C_21_H_52_O_6_Si_5_	284840388	1.8
11	D-Ribose, 4TMS	22.26	C_17_H_42_O_5_Si_4_	137797020	0.9
12	D-Mannitol, 6TMS	22.68	C_24_H_62_O_6_Si_6_	176044567	1.1
13	Ethyl-alpha-D-glucopyranoside, 4TMS	22.85	C_20_H_48_O_6_Si_4_	485941032	3.1
14	Gallic acid, 4TMS	23.07	C_19_H_38_O_5_Si_4_	231050954	1.5
15	D-Gluconic acid, 6TMS	24.9	C_24_H_60_O_7_Si_6_	93522212	0.6
16	Palmitic acid, TMS	25.31	C_19_H_40_O_2_Si	183550036	1.2
17	1,2,3,4,5,6-Hexa-O-trimethelsilyl-myo-inositol	27.5	C_24_H_60_O_6_Si_6_	1379611112	8.9
18	Phytol, TMS	28.56	C_23_H_48_OSi	169019263	1.1
19	9,12-Octadecadienoic acid (Z,Z), TMS	29.16	C_21_H_40_O_2_Si	142435170	0.9
20	alpha-Linolenic acid, TMS	29.29	C_21_H_38_O_2_Si	210225278	1.4
21	Stearic acid, TMS	29.67	C_21_H_44_O_2_Si	106003067	0.7
22	Galactinol, 9TMS	30.56	C_39_H_94_O_11_Si_9_	33296887	0.2
23	Glyceryl-glycoside, 6TMS	31.3	C_27_H_66_O_8_Si_6_	32377994	0.2
24	D-(+)-Galacturonic acid, 5TMS	32	C_21_H_50_O_7_Si_5_	42093264	0.3
25	3-alpha-Mannobiose, 8TMS (isomer 1)	33.41	C_36_H_86_O_11_Si_8_	151874014	1.0
26	Sucrose, 8TMS	34.74	C_36_H_86_O_11_Si_8_	237500188	1.5
27	Epicatechin, 5TMS	36.47	C_30_H_54_O_6_Si_5_	187679647	1.2
28	Epigallocatechin, 6TMS	36.98	C_33_H_62_O_7_Si_6_	28350263	0.2
29	Catechin (2R-E), 5TMS derivative	37.49	C_30_H_54_O_6_Si_5_	183518342	1.2
30	5-O-Coumaroyl-D-quinic acid, 5TMS	37.56	C_31_H_58_O_8_Si_5_	246536824	1.6
31	Maltose, 8TMS (isomer 1)	38.03	C_36_H_86_O_11_Si_8_	391816235	2.5
32	Tetrahydrocannabinolic acid A (THCA-A), TMS	38.23	C_28_H_46_O_4_Si_2_	51005267	0.3
33	Chlorogenic acid, 6TMS	39.05	C_34_H_66_O_9_Si_6_	237351823	1.5
34	Stigmasterol, TMS	41.15	C_32_H_56_OSi	29985323	0.2
35	Stigmast-5-ene, 3-beta-(trimethylsiloxy)-, (24S)-	41.96	C_32_H_58_OSi	294152074	1.9
36	1,5-Anhydroglucitol, 4TMS	42.36	C_18_H_44_O_5_Si_4_	295770539	1.9
37	Lupeol, TMS	42.75	C_33_H_58_OSi	46691537	0.3
				7992607793	51.4

**Table 2:** Peak identification for the chromatogram of silylated *S. pittieriana* extract, which is shown in Supplementary Figure S2.

No.	Silylated Derivative	Retention Time (RT, min)	Formula (DB)	Area	Relative Concentration (%)
1	Benzoic acid, TMS	6.74	C_10_H_14_O_2_Si	9434738	0.1
2	Glycerol, 3TMS	7.29	C_12_H_32_O_3_Si_3_	55769025	0.9
3	Erythrono-1,4-lactone, 2TMS	9.46	C_10_H_22_O_4_Si_2_	6874007	0.1
4	meso-Erythritol, 4TMS	12.23	C_16_H_42_O_4_Si_4_	53487190	0.8
5	2-Amino-1-phenylethanol, TMS	13.35	C_11_H_19_NOSi	78884262	1.2
6	1,3,5-Benzetriol, 3TMS	14.66	C_15_H_30_O_3_Si_3_	554013402	8.7
7	Levoglucosan, 3TMS	16.18	C_15_H_34_O_5_Si_3_	74548119	1.2
8	1,3-Dihydroxyacetone dimer, 4TMS	17.46	C_18_H_44_O_6_Si_4_	70194953	1.1
9	L-(-)-Sorbofuranose, 5TMS	18.58	C_21_H_52_O_6_Si_5_	7063162	0.1
10	D-Psicofuranose, 5TMS (isomer 2)	19.71	C_21_H_52_O_6_Si_5_	206102805	3.2
11	beta-Lyxopyranose, 4TMS	20.01	C_17_H_42_O_5_Si_4_	341679723	5.4
12	Methyl-alpha-D-glucofuranoside, 4TMS	20.49	C_19_H_46_O_6_Si_4_	585207154	9.2
13	Quinic acid, 5TMS*	20.84	C_22_H_52_O_6_Si_5_	342405065	5.4
14	1,5-Anhydroglucitol, 4TMS derivative	21.7	C_18_H_44_O_5_Si_4_	77502599	1.2
15	alpha-D-(-)-Lyxopyranose, 4TMS	21.93	C_17_H_42_O_5_Si_4_	190111264	3.0
16	Gallic acid, 4TMS	23.19	C_19_H_38_O_5_Si_4_	7963547	0.1
17	D-Allofuranose, 5TMS	24.42	C_21_H_52_O_6_Si_5_	152662261	2.4
18	D-(+)-Talofuranose, 5TMS (isomer 1)	24.81	C_21_H_52_O_6_Si_5_	73045683	1.1
19	Palmitic acid, TMS	25.19	C_19_H_40_O_2_Si	132917257	2.1
20	D-Xylopyranose, 4TMS	26.73	C_17_H_42_O_5_Si_4_	6915739	0.1
21	1,2,3,4,5,6-Hexa-O-trimethelsilyl-myo-inositol	27.48	C_24_H_60_O_6_Si_6_	511382622	8.0
22	Phytol, TMS	28.53	C_23_H_48_OSi	90203686	1.4
23	9,12-Octadecadienoic acid (Z,Z)-, TMS	29.12	C_21_H_40_O_2_Si	31352387	0.5
24	alpha-Linolenic acid, TMS	29.26	C_21_H_38_O_2_Si	59691843	0.9
25	Stearic acid, TMS	29.64	C_21_H_44_O_2_Si	17120338	0.3
26	Glyceryl-glycoside TMS	31.3	C_27_H_66_O_8_Si_6_	9908413	0.2
27	1-Monopalmitin, 2TMS	33.8	C_25_H_54_O_4_Si_2_	16264915	0.3
28	D-(+)-Turanose, 8TMS	34.21	C_36_H_86_O_11_Si_8_	9168678	0.1
29	Sucrose, 8TMS	34.71	C_36_H_86_O_11_Si_8_	51634160	0.8
30	Catechin (2R-E)-, 5TMS	37.73	C_30_H_54_O_6_Si_5_	113798634	1.8
31	Cholest-2-eno [2,3-b]indole, 1′-methyl-5′-methoxy-	39.1	C_35_H_53_NO	79490945	1.2
32	Campesterol, TMS	40.72	C_31_H_56_OSi	8562042	0.1
33	Stigmasterol, TMS	41.12	C_32_H_56_OSi	15449063	0.2
34	gamma-Sitosterol	41.56	C_29_H_50_O	17799230	0.3
35	Stigmast-5-ene, 3-beta-(trimethylsiloxy)-, (24S)-	41.95	C_32_H_58_OSi	162188141	2.5
36	Lup-20(29)-en-28-al, 3-(trimethylsilyl)oxy, (3-beta)-	42.49	C_33_H_56_O_2_Si	6824836	0.1
37	alpha-Amyrin, TMS	42.63	C_33_H_58_Osi	6959812	0.1
38	Oleanolic acid, 2TMS	45.39	C_36_H_64_O_3_Si_2_	47379205	0.7
				6374070839	67.2

**Table 3:** Antifungal activity of ethanolic extracts from *S. chocoana* and *S. pittieriana*.

*Sloanea* Extract	GM–MIC ^a^ (µg/mL)				
	*Candida albicans* ATCC 10231 ^b^	*Candida auris* Ca17 (L25) ^c^	*Candida glabrata* LMDM 34 (L7) ^c^	*Candida krusei* ATCC 6258 (L6) ^b^	*Candida tropicalis* ATCC 200956 ^b^
*S. chocoana*	16	64	32	128	128
*S. pittieriana*	4	32	32	128	ND
Amphotericin B	-	-	-	0.630	-
Itraconazole	-	-	-	0.125	-

^a^ Geometric mean–minimum inhibitory concentration. Three independent assays were performed. ^b^ Laboratory reference *Candida* species. ^c^ Clinical isolates of *Candida* species. ND: Not determined.

**Table 4:** Antioxidant activity of ethanolic extracts from *S. chocoana* and *S. pittieriana*.

*Sloanea* Extract	MEAN ± SEM (n = 3)	
	DPPH ^a^ Radical Scavenging Activity (%)	TEAC ^b^
*S. chocoana*	46.1 ± 0.0	3.7 ± 0.0
*S. pittieriana*	43.7 ± 0.0	2.0 ± 0.0

^a^ 1,1-diphenyl-2-picrylhydrazyl. ^b^ Trolox equivalent antioxidant capacity. Mean ± standard error from three independent assays was used to express the antioxidant activity.

**Table 5:** In vitro measurements of photoprotective properties of ethanolic extracts from *S. chocoana* and *S. pittieriana*.

*Sloanea* Extract	MEAN ± SEM (*n* = 3)				
	SPF ^a^	λc ^b^	UVA/UVB Ratio	Transmission of Erythema (%)	Transmission of Pigmentation (%)
*S. chocoana*	31.0 ± 0.2 ^c^	393.98 ± 0.0 ^d^	1.5 ± 0.0	0.0 ± 0.0	0.9 ± 0.0
*S. pittieriana*	30.0 ± 0.1 ^c^	337.81 ± 0.1	1.2 ± 0.0	0.0 ± 0.0	2.8 ± 0.1
Sunscreen ^e^	14.4 ± 0.3 ^f^	393.19 ± 0.0 ^d^	0.3 ± 0.0	1.3 ± 0.1	24.9 ± 0.5

^a^ Sun protective factor. ^b^ Critical wavelength (nm). ^c^ Extract meets the criteria of high UV protection. ^d^ UVA long wavelength sunscreen. ^e^ Commercial sunscreen with vitamin E and *Aloe vera*. ^f^ Extract meets the criteria of low UV protection. Commercial sunscreen was prepared at a concentration of 0.75 mg/mL. Mean ± standard error from three independent assays was used to express the UV-protective activity.

## Data Availability

Data presented in this study are contained within the article and Supplementary Materials.

## Supplementary Materials

The following supporting information can be downloaded at: https://www.mdpi.com/article/10.3390/plants12233953/s1, Figure S1. Representative GC–MS chromatogram of silylated ethanolic extract from *Sloanea chocoana* leaves. Figure S2. Representative GC–MS chromatogram of silylated ethanolic extract from *Sloanea pittieriana* leaves. Table S1. Some criteria for consideration of in vitro photoprotective potential.
