# Supplementary material for: Sloanea chocoana and S. pittieriana (Elaeocarpaceae): Chemical and Biological Studies of Ethanolic Extracts and Skincare Properties
Source: Plants (Basel). 2023 Nov 24;12(23):3953. doi: 10.3390/plants12233953 (PMC10708535; doi:10.3390/plants12233953)
Supplement: Supplementary file 1 [file plants-12-03953-s001.zip › plants-2699943-supplementary.pdf]

Supplementary Information

Sloanea chocoana and *S. pittieriana* (Elaeocarpaceae): Chemical and Biological Studies of Ethanolic Extracts and Skincare Properties

Patricia Quintero-Rincón [1,2], Nayive Pino-Benítez [1,*], Elkin Galeano [3], Cris Rojo-Uribe [4], Ana C. Mesa-Arango [4] and Oscar A. Flórez-Acosta [2]

[1] Natural Products Group, Universidad Tecnológica del Chocó, Quibdo 270002, Colombia; patriciaquintero@gmail.com

[2] Research Group Design and Formulation of Medicines, Cosmetics, and Related, Faculty of Pharmaceutical and Food Sciences, Universidad de Antioquia, Medellin 050010, Colombia; oscar.florez@udea.edu.co

[3] Bioactive Substances Research Group, Faculty of Pharmaceutical and Food Sciences, Universidad de Antioquia, Medellin 050010, Colombia; elkin.galeano@udea.edu.co

[4] Dermatological Research Group, Faculty of Medicine, Universidad de Antioquia, Medellin 050010, Colombia; cris.rojo@udea.edu.co (C.R.-U.); ana.mesa@udea.edu.co (A.C.M.-A.)

* Correspondence: nayivepino@gmail.com; Tel.: +57-310-4559617

Legends:

Supplementary Figure S1. Representative GC-MS chromatogram of silylated ethanolic extract from *Sloanea chocoana* leaves.

Supplementary Figure S2. Representative GC-MS chromatogram of silylated ethanolic extract from *Sloanea pittieriana* leaves.

Supplementary Table S1. Some criteria for consideration of *in vitro* photoprotective potential.

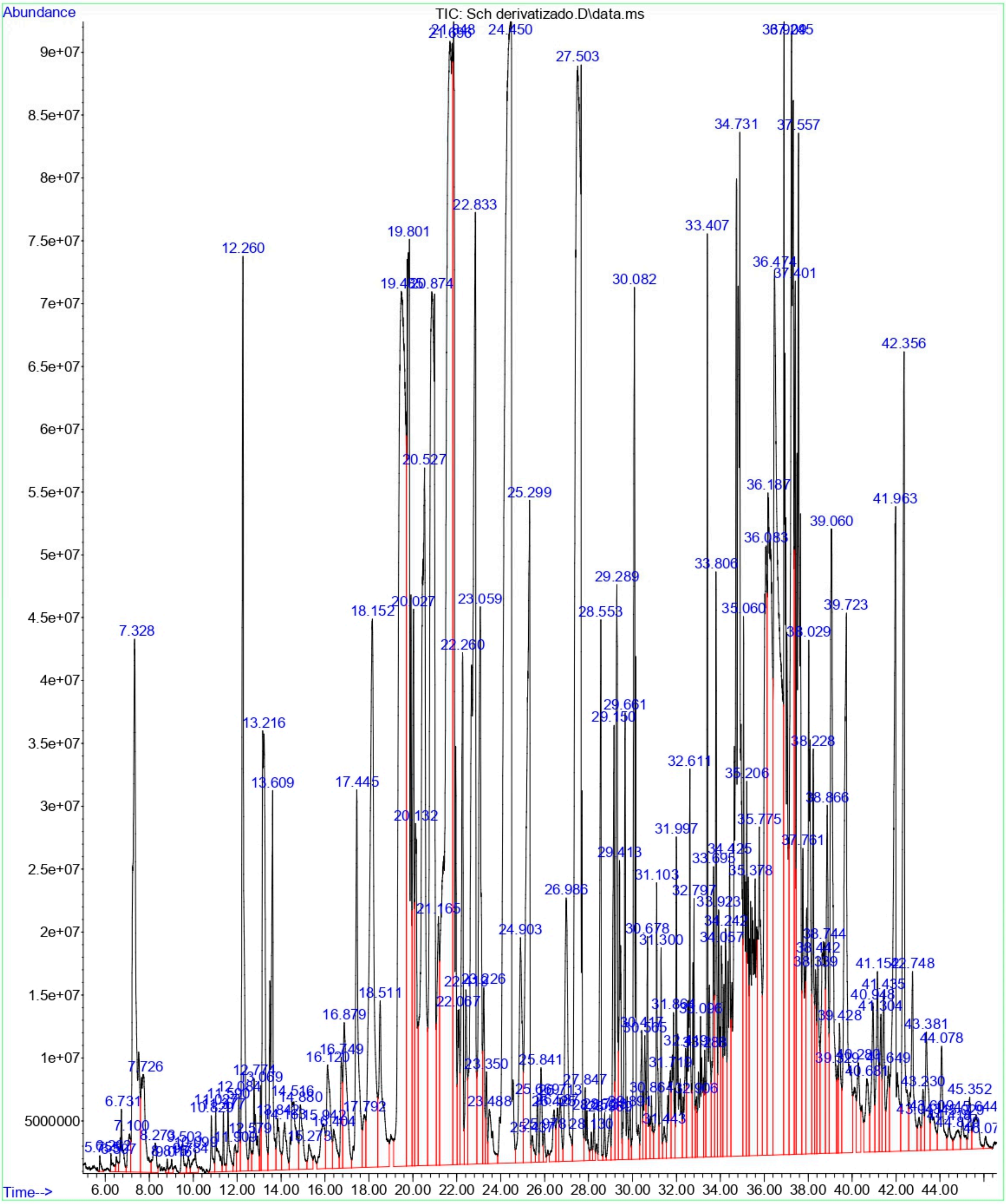

Supplementary Figure S1. Representative GC-MS chromatogram of silylated ethanolic extract from *Sloanea chocoana* leaves.

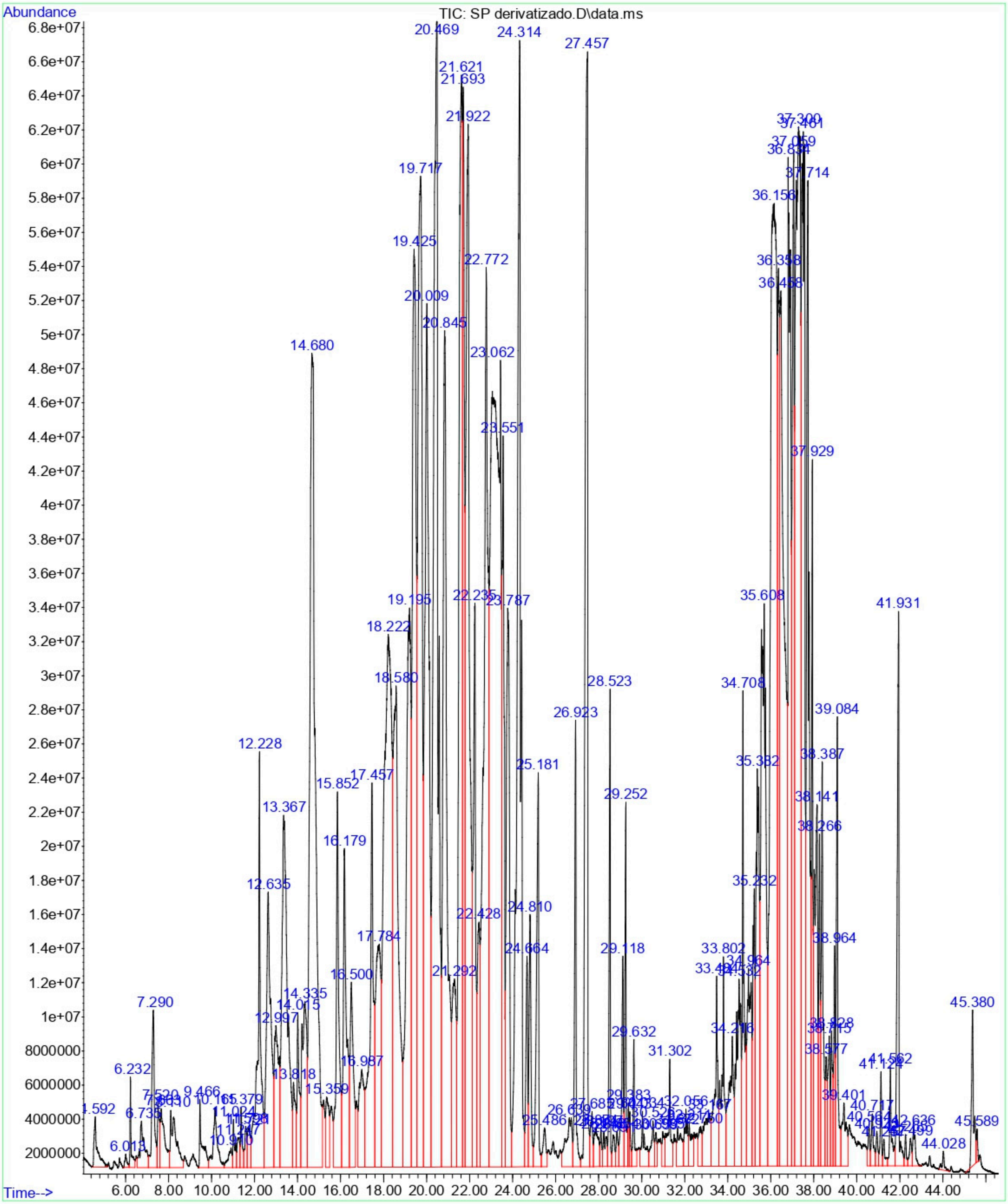

Supplementary Figure S2. Representative GC-MS chromatogram of silylated ethanolic extract from *Sloanea pittieriana* leaves.

Supplementary Table S1. Some criteria for consideration of in vitro photoprotective potential.

No.	Evaluated Parameter	General Considerations [a]	UV Protection Criteria
1	Sun Protective Factor (SPF)	Absorbance is measured in the wavelength range 290 to 320 nm (UVB), using an optical path of 5.0 cm. To obtain the SPF values, the Mansur equation should be applied: $\mathrm{SPF} = \mathrm{CF} \times \sum_{290\ \mathrm{nm}}^{320\ \mathrm{nm}} \mathrm{EE}\ (\lambda) \times \mathrm{I}(\lambda) \times \mathrm{Abs}\ (\lambda)$	SPF 2-15 (low protection)<br>SPF 15-30 (moderate protection)<br>SPF 30-50 (high protection)<br>SPF >50 (maximum protection)
2	Critical wavelength (λc)	Absorbance is measured in the wavelength range of 290 to 400 nm (UVA and UVB), using an optical path of 1.0 cm. To determine λc values, the following equation is applied: $\int_{290\ \mathrm{nm}}^{\lambda c} \mathrm{A}\ (\lambda) d\lambda = 0.9 \int_{290\ \mathrm{nm}}^{400\ \mathrm{nm}} \mathrm{A}(\lambda) d\lambda$	According to the FDA, a product is considered to have broad-spectrum protection when its minimum critical wavelength is ≥370 nm
3	UVA/UVB ratio	Absorbance is measured in the wavelength range of 290 to 400 nm (UVA and UVB), using an optical path of 5.0 cm. To determine the UVA/UVB ratio, the following equation is applied: $\frac{\mathrm{UVA}}{\mathrm{UVB}} = \frac{\sum_{320\ \mathrm{nm}}^{400\ \mathrm{nm}} \mathrm{A}(\lambda), \mathrm{d}(\lambda)}{\sum_{290\ \mathrm{nm}}^{320\ \mathrm{nm}} \mathrm{A}(\lambda), \mathrm{d}(\lambda)}$	The star rating system indicates that 0.0 to <0.2 is too low for UVA protection (-), 0.2 to <0.4 is a moderate protector (*), 0.4 to <0.6 is a good protector (**), 0.6 to <0.8 is a superior protector (***), and 0.8 to ≥0.8 is a maximum protector (****)
4	Transmission of erythema (%)	Absorbance is measured in the wavelength range of 292 to 372 nm (UVA and UVB), using an optical path of 5.0 cm. It is necessary calculates transmission (T) using A = 1/ T = - log T. Then, the following equation is applied: $\text{Transmission of erythema (\%)} = \frac{\mathrm{Ee}}{\sum \mathrm{Fe}} = \sum \frac{(\mathrm{T} \times \mathrm{Fe})}{\sum \mathrm{Fe}}$	UV transmission range (%) [b]<br>Sunscreen, <1<br>Extra protection, 1-6<br>Standard tan, 6-12<br>Quick tan, 10-18
5	Transmission of pigmentation (%)	Absorbance is measured in the wavelength range of 292 to 372 nm (UVA and UVB), using an optical path of 5.0 cm. It is necessary calculates transmission (T) using A = 1/ T = - log T. Then, the following equation is applied: $\text{Transmission of pigmentation (\%)} = \frac{\mathrm{Ep}}{\sum \mathrm{Fp}} = \sum \frac{(\mathrm{T} \times \mathrm{Fp})}{\sum \mathrm{Fp}}$	UV transmission range (%) [b]<br>Sunscreen, 3-40<br>Extra protection, 42-86<br>Standard tan, 45-86<br>Quick tan, 45-86

[a] A description detailed for each evaluated parameter is found in Quintero-Rincón et al. [13] and Caballero-Gallardo et al. [25].
[b] These values must be as low as possible to be considered sunscreen.
